# Behavioral Effects of Continuously Administered Bergamot Essential Oil on Mice With Partial Sciatic Nerve Ligation

**DOI:** 10.3389/fphar.2020.01310

**Published:** 2020-08-21

**Authors:** Kengo Hamamura, Soh Katsuyama, Takaaki Komatsu, Damiana Scuteri, Giacinto Bagetta, Kosuke Aritake, Tsukasa Sakurada

**Affiliations:** ^1^Laboratory of Chemical Pharmacology, Faculty of Pharmaceutical Sciences, Daiichi University of Pharmacy, Fukuoka, Japan; ^2^Center for Clinical Pharmacology and Pharmaceutics, Nihon Pharmaceutical University, Saitama, Japan; ^3^Drug Analysis Laboratory, Faculty of Pharmaceutical Sciences, Daiichi University of Pharmacy, Fukuoka, Japan; ^4^Preclinical and Translational Pharmacology, Department of Pharmacy, Health Science and Nutrition, University of Calabria, Cosenza, Italy; ^5^Center for Supporting Pharmaceutical Education, Faculty of Pharmaceutical Sciences, Daiichi University of Pharmacy, Fukuoka, Japan

**Keywords:** bergamot essential oil, neuropathic pain, partial sciatic nerve ligation, osmotic pump, double activity monitoring system^®^

## Abstract

Neuropathic pain is an intractable chronic pain condition that is mainly caused by allodynia. We had previously reported that intra-plantar administration of bergamot essential oil (BEO) containing an aromatic compound significantly suppressed partial sciatic nerve ligation (PSNL)-induced mechanical allodynia *via* opioid mu receptors in mice. However, it has also been reported that the inhalation of BEO reduced formalin-induced nociceptive responses. Therefore, we aimed to elucidate whether the analgesic action of BEO is mediated by olfactory stimulation through volatile components. In the current study, BEO was continuously administered with an osmotic pump during PSNL surgery, and the effects on mice behavior were examined pharmacologically using a double activity monitoring system, which can detect two-dimensional planar motion in a cage with an infrared beam sensor as well as active motion with a running wheel. Here, we report that the two-dimensional planar activity significantly increased in mice with PSNL in the light phase (from 8 o’clock to 20 o’clock) but not in the dark phase (from 20 o’clock to 8 o’clock) from the second day after surgery. However, this increase was not observed when BEO was continuously administered. The effect of BEO on the two-dimensional planar counts in mice with PSNL was antagonized by naloxone hydrochloride. Regarding the running wheel activity, the number of rotations decreased by PSNL in the dark phase from the 8th day after surgery. However, this was not apparent with BEO use. The effect of BEO on the number of rotations was also antagonized by naloxone hydrochloride. Furthermore, inhalation of BEO in PSNL mice did not affect mechanical allodynia or the two-dimensional planar motion or running wheel activities. These findings indicate that BEO exhibits an analgesic action, which is mediated by opioid receptors and not by the olfactory system.

## Introduction

Neuropathic pain is a chronic condition that occurs after nerve compression due to cancer, diabetes, herpes virus infection, and autoimmune diseases (Woolf and Mannion, 1999). Currently, millions of patients worldwide endure neuropathic pain (Tsuda et al., 2005). One troubling and characteristic symptom of neuropathic pain is hypersensitivity to usually harmless stimuli, a condition known as “tactile allodynia,” which is often resistant to NSAIDs and opioids (Backonja and Glanzman, 2003). Various models have been devised to reproduce disease-like conditions in rodents, such as diabetic neuropathy, chemotherapy-induced neuropathic pain, antiretroviral drug-induced neuropathy, and spinal and peripheral nerve damage. In recent years, it has become clear that chronic pain can affect cognitive behavior in animal models just as it does in humans (Guida et al., 2020). Therefore, the establishment of treatments for neuropathic pain is an important issue in terms of reducing anxiety and depression.

Aromatherapy refers to therapies that use essential oil or plant-derived fragrances to prevent illnesses, improve mental and physical health and relaxation, and relieve stress. Among the essential oils, bergamot essential oil (BEO) is obtained by cold pressing the epicarp and part of the mesocarp of the bergamot fruit (*Citrus bergamia* Risso et Poiteau) (Moufida and Marzouk, 2003). BEO is listed in Farmacopea Ufficiale Italiana (1991~) and is used in the pharmaceutical industry, mainly in dentistry, ophthalmology, and dermatology. Recently, several reports showed that the use of aromatherapy massage with various essential oils including BEO could relieve anxiety (Wilkinson et al., 2007; Seyyed-Rasooli et al., 2016), depressions (Wilkinson et al., 2007), and the perception of pain (Nasiri et al., 2016; Seyyed-Rasooli et al., 2016; Gok Metin et al., 2017).

Regarding the pain area, we had reported that the capsaicin-induced nociceptive response was significantly reduced by the intra-plantar injection of BEO in mice (Kuwahata et al., 2009). Next, we had reported that the opioid receptor antagonist naloxone hydrochloride significantly reversed the inhibitory effects of BEO on the capsaicin-induced behavioral response (Katsuyama et al., 2011). Next, we had performed a 2% formalin test as another nociceptive pain model mice. Plantar subcutaneous injection of 2% formalin caused biphasic (phases I and II) nociceptive behavior consisting of licking and biting. We reported that plantar subcutaneous injection of BEO reduced both the first and late phases of the formalin-induced licking and biting responses (Katsuyama et al., 2015). This inhibitory effect of BEO on the formalin-induced behavioral response was also antagonized by naloxone hydrochloride (Katsuyama et al., 2015). Therefore, BEO can be efficacious in nociceptive pain, and help to suppress partial sciatic nerve ligation (PSNL) mouse-induced allodynia (Komatsu et al., 2018). The inhibitory effect of BEO on PSNL-induced allodynia is also antagonized by naloxone methiodide (Komatsu et al., 2018).

BEO consists of volatile fractions (93–96% of the total) of monoterpene and sesquiterpene hydrocarbons (such as limonene) and oxygenated derivatives (such as linalool) and non-volatile fractions (4–7% of the total) of waxes, polymethoxylated flavones, coumarins, and psoralens, such as bergapten (5-methoxypsoralen) and bergamottine (5-geranyloxypsoralen) (Mondello et al., 1993; Dugo et al., 2000). Regarding the pharmacological action of scents, BEO inhalation was found to produce anxiolytic-like behavior (Brederson et al., 2013). More recently, BEO inhalation has been reported to reduce the behavioral signs of formalin-induced nociception in a dose-dependent manner (Scuteri et al., 2018). However, it was unclear whether the compound contained in BEO or its volatile component was important for anti-allodynic action. Therefore, we aimed to elucidate whether the analgesic action of BEO is mediated by olfactory stimulation by volatile components. In this study, we used PSNL mice, a neuropathic pain model, and we set up two experimental systems to examine behavior pharmacologically using a double activity monitoring system^®^, which can detect two-dimensional planar motion in a cage with an infrared beam sensor, as well as active motion with a running wheel. First, BEO was continuously administered subcutaneously with an osmotic pump to eliminate the effects of scent as much as possible. Second, we investigated the effects of BEO inhalation on pain-related activities.

## Materials and Methods

### Animals

Four-week-old, male *ddY*-strain mice, weighing an average of 24 g (Japan SLC, Inc., Hamamatsu, Japan) were housed in groups (from 6 to 10 per cage) in a light-controlled room (illuminated from 8 o’clock to 20 o’clock) at 24 ± 1°C and 60 ± 10% humidity with free access to food (LabDiet 5L37, Japan SLC, Inc.) and water. Mice were acclimatized to the lighting conditions for 1 week. All experiments were performed following the approval of the Ethics Committee for Animal Experiments at Daiichi University of Pharmacy (Examination number: H30-006, approval number: 29004) and according to the National Institutes of Health Guide for the Care and Use of Laboratory Animals (Zimmermann, 1983). Every effort was made to minimize the number of animals used and any suffering in the experiments.

### Partial Sciatic Nerve Ligation (PSNL)

The neuropathic pain model was created by ligating part of the sciatic nerve of 5-week-old male *ddY*-strain mice. Under anesthesia with isoflurane (2.0%, FUJIFILM Wako Pure Chemical Corporation, Osaka, Japan), the sciatic nerve in the upper right thigh was exposed, and about half of the sciatic nerve was strongly ligated using 4-0 silk thread (Kusunose et al., 2010). Mice in which the sciatic nerve was not ligated formed the sham-operated group.

### Double Activity Monitoring System^®^

**Figure 1:**
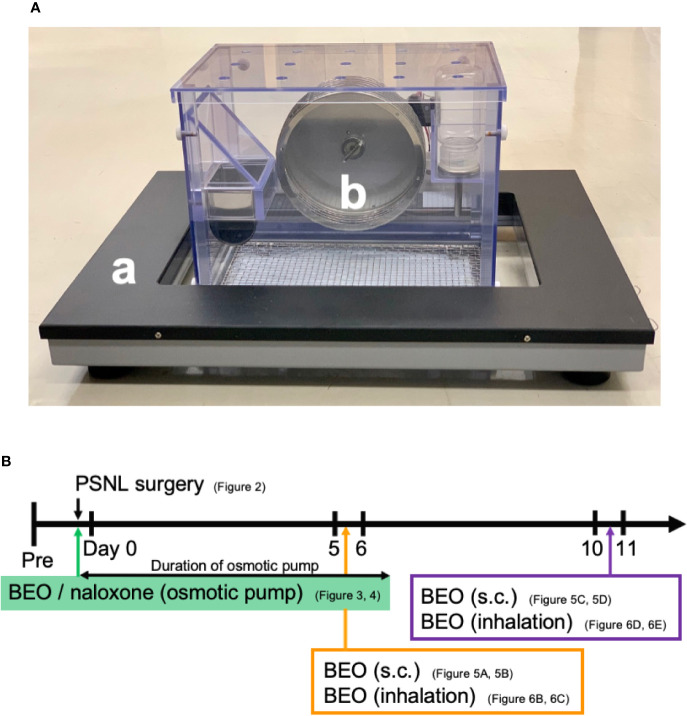
**(A)** Behavior was analyzed using a double activity monitoring system^®^. (a) The planar activity was measured using an animal movement analyzing system. (b) The running wheel activity was measured using a cage with a rotating basket. **(B)** The scheme of experiments. Each experiment was conducted independently.

Behavior was analyzed using a double activity monitoring system^®^ (ShinFactory, Fukuoka, Japan) (**Figure 1A**), which can detect two-dimensional planar motion in a cage with an infrared beam sensor as well as active motion with a running wheel every 15 min. The planar activity was measured using an animal movement analyzing system (ACTIMO-100, ShinFactory), which consists of a rectangular enclosure (30 × 20 cm) with a side wall equipped with photo sensors at 1.7 cm intervals. The running wheel activity was measured using a cage with a rotating basket (ACTIMO-RWM, ShinFactory). As for the results, the time zone with a white background on the horizontal axis is the light period from 8 o’clock to 20 o’clock, and with the gray background is the dark period from 20 o’clock to the next day’s 8 o’clock. The behavior measurement started the day before the PSNL operation, and the operation day was set to Day 0. The mice were then observed for 14 days. The theoretical duration of the osmotic pump, which was 1 week, is shown with an orange background.

### Assessment of Mechanical Allodynia by Von Frey Test

To assess mechanical allodynia, the mice were individually placed in a plastic animal chamber (internal dimensions 90 × 90 × 140 mm, Ugo Basile, Gemonio, Italy) on a stainless-steel mesh floor and habituated for 0.5 h to allow for acclimatization to the new environment. Calibrated von Frey filaments (pressure stimulus 0.40 g, Natsume Seisakusho Co., Ltd., Tokyo, Japan) were then applied to the right plantar surfaces of the hind paws of the mice. The paw withdrawal threshold was evaluated using the up-down method (Seltzer et al., 1990; Kusunose et al., 2010).

### Drug Administration Using an Osmotic Pump

BEO was kindly provided from “Capua Company1880 S.r.l.,” Campo Calabro, Reggio Calabria (Italy). According to the chromatographic analysis provided in the certificate of analysis, this batch of BEO contained: D-limonene (39.60%), linalyl acetate (31.09%), and linalool (9.55%). Jojoba wax (vehicle of BEO) was provided by “Company Farmalabor,” Canosa of Puglia (Italy). Naloxone hydrochloride (Sigma-Aldrich, MO, USA) was dissolved in physiological saline (0.9% aqueous solution of sodium chloride, Nacalai Tesque, Kyoto, Japan). An Alzet^®^ model 1007D mini osmotic pump (100 μl, 0.5 μl/h, 1 week sustained type, Durect Corporation Cupertino, CA, USA) containing either BEO (100 μl of stock solution) alone, BEO (100 μl of stock solution) and naloxone hydrochloride (1 mg/100 μl), or jojoba wax (100 μl of stock solution) alone were surgically implanted under the back of mice anesthetized with isoflurane at the same time as PSNL (**Figure 1B**). The skin incision was closed with surgical sutures. The mice were immediately returned to the behavioral cage.

### BEO Administration by Inhalation

BEO (0.04, 0.4, 4, 40, 400 μl/cage) was diluted with triethyl citrate to a total volume of 400 μl, and 100 μl each was dropped onto four filter paper discs attached to the four corners of the glass cage (30  × 60 × 34 cm; 61.2 L). The cage was filled with the vaporized solution by diffusion for 60 min. PSNL mice were then placed in the glass cage and they inhaled this for 60 min. After that, mice were removed from the glass cages, and a behavioral analysis was performed. In addition, when examining the effect on two-dimensional planar motion, inhalation of BEO in PSNL mice was performed from 10 o’clock to 11 o’clock on the 5th postoperative day. When examining the effect on the running wheel activity, inhalation of BEO by PSNL mice was performed from 18 o’clock to 19 o’clock on the 11th postoperative day.

### Immunohistochemistry

Olfactory bulb samples were fixed with 4% paraformaldehyde (powder, Nacalai Tesque). The fixed samples were dehydrated with 30% sucrose (Nacalai Tesque) and embedded in O.C.T. compound (Sakura Finetek Japan Co. Ltd., Tokyo, Japan). Sections (20 μm thick) were antigen-activated with Histo VT One (Nacalai Tesque) for 20 min at 80°C. The steps after blocking were performed according to the protocol of the M.O.M. immunodetection kit (FMK-2201; Vector Laboratories, CA, USA). Anti-c-fos antibody (Santa Cruz Biotechnology, CA, USA) was used as the primary antibody. The sections were mounted with mounting medium (Vectashield Hard Set Mounting Medium with DAPI; Vector Laboratories). Fluorescent images were obtained with a fluorescence microscope (BZ-X810; Keyence Corporation, Osaka, Japan).

### Statistical Analysis

Results are presented as mean ± standard error of the mean (S. E.M). Statistical differences were analyzed using the Student’s t-test for two-group comparisons, and a one-way analysis of variance with Tukey’s test for multiple-group comparisons. Statistical analysis was performed with Excel Statistics (Social Survey Research Information Co., Ltd., Tokyo, Japan). A p-value of <0.05 was considered statistically significant.

## Results

### Two-Dimensional Planar Behavior Counts in PSNL Mice Increased in the Light Phase

**Figure 2:**
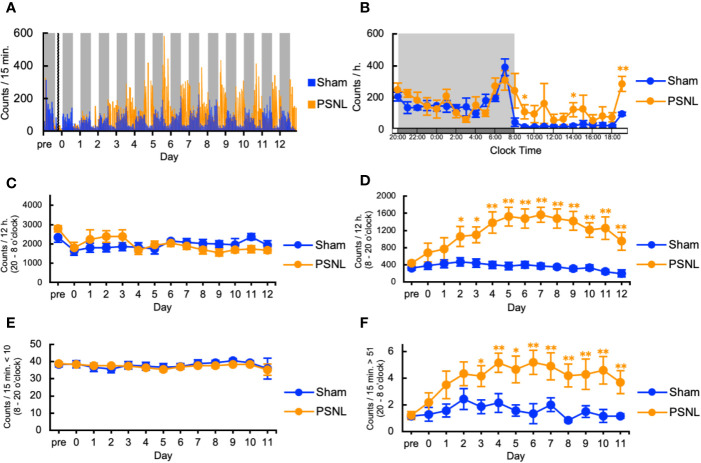
Assessment of two-dimensional planar motion in partial sciatic nerve ligation (PSNL) mice. **(A)** Representative data of two-dimensional planar motion in PSNL mice every 15 min. **(B)** Circadian variation of two-dimensional planar behavior in PSNL mice on the 7th postoperative day. **(C, D)** Assessment of two-dimensional planar motion in PSNL mice during the dark period (20 o’clock–8 o’clock) **(C)**, and light period (8 o’clock–20 o’clock) **(D)**. **(E, F)** Assessment of two-dimensional planar immobility time (count per 15 min: 10 or less) **(E)**, and mobility time (count per 15 min: over 51) **(F)** in PSNL mice during the light period. **(B–F)** Values are the means ± S.E.M. (Sham: n = 7, PSNL: n = 9). **(B, D, F)** *P < 0.05; **P < 0.01, compared with the values at corresponding time points (Student’s t-test).

First, we investigated the behavioral phenotype of PSNL mice. In the two-dimensional planar activity, representative data showed that the number of counts in mice with PSNL increased only in the light phase (from 8 o’clock to 20 o’clock) but not in the dark phase (from 20 o’clock to next 8 o’clock) from the second day after surgery (**Figure 2A**). On the 7^th^ postoperative day, when allodynia was the most intense (Kusunose et al., 2010), a comparison of the circadian changes in the two-dimensional planar activity revealed an increase at any time during the light period in PSNL mice (**Figure 2B**). Total counts of the two-dimensional planar activity during the dark phase did not change (**Figure 2C**). The total number of two-dimensional planar counts during the light phase of PSNL mice increased significantly from day 2 post-surgery, reaching its maximum at day 7 post-surgery (Sham: 369.83 ± 69.41, PSNL: 1,566.71 ± 175.23) (**Figure 2D**). To examine whether this was caused by a decrease in immobility time, a threshold value was set at 10 or less per 15 min, and the total number was calculated. As a result, immobility time in the light phase did not change between the PSNL and sham-operated groups (**Figure 2E**). The threshold was set to be more than 51 every 15 min to investigate whether it was due to an increase in the time. This showed that the number of counts significantly increased from day 3 post-surgery in PSNL mice and reached its maximum at day 6 post-surgery (Sham: 1.86 ± 0.51, PSNL: 5.20 ± 0.90) (**Figure 2F**). These results indicate that PSNL mice have a behavioral phenotype that changes a lot once they start to move during the light period.

### Increased Counts by PSNL in the Light Phase Was Abolished by Continuously Administered BEO and Antagonized by Naloxone Hydrochloride

**Figure 3:**
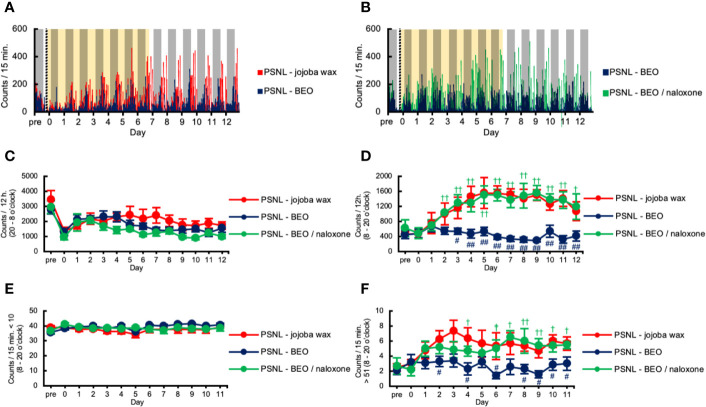
Assessment of two-dimensional planar motion in PSNL mice with continuous subcutaneous administration of bergamot essential oil (BEO) and naloxone hydrochloride using an osmotic pump. **(A, B)** Representative data of two-dimensional planar motion in PSNL mice with continuous subcutaneous administration of jojoba-wax and BEO **(A)**, and BEO and BEO/naloxone **(B)** every 15 min using an osmotic pump. **(C, D)** Assessment of two-dimensional planar motion in PSNL mice with continuous subcutaneous administration of jojoba-wax, BEO, or BEO/naloxone using an osmotic pump during the dark period (20 o’clock–8 o’clock) **(C)** and light period (8 o’clock–20 o’clock) **(D)**. **(E, F)** Assessment of two-dimensional planar immobility time (count per 15 min: 10 or less) **(E)**, and mobility time (count per 15 min: over 51) **(F)** in PSNL mice with continuous subcutaneous administration of jojoba-wax, BEO and BEO/naloxone using an osmotic pump during the light period. **(C–F)** Values are the means ± S.E.M. (PSNL-jojoba wax: n = 6, PSNL-BEO: n = 9, PSNL-BEO/naloxone: n = 6). **(D, F)** ^#^P < 0.05; ^##^P < 0.01, compared with PSNL-jojoba wax and PSNL-BEO at corresponding time points. ^†^P < 0.05; ^††^P < 0.01, compared with PSNL-BEO and PSNL-BEO/naloxone at corresponding time points (one-way analysis of variance with Tukey’s test).

We investigated the effect of continuous administration of BEO using an osmotic pump on the two-dimensional planar activity in PSNL mice. Representative data showed that the increased counts produced by PSNL were abolished by continuously administered BEO in the light phase (**Figure 3A**). The effect of BEO on the two-dimensional planar counts in mice with PSNL was antagonized by naloxone hydrochloride (**Figure 3B**). Continuous administration of BEO and naloxone hydrochloride did not affect the total counts of two-dimensional planar activity during the dark phase (**Figure 3C**). The increase in the two-dimensional planar counts of PSNL-jojoba wax mice during the light phase was significantly decreased from day 3 post-surgery with continuous administration of BEO, and continued until the day 12 post-surgery at the end of the measurement (**Figure 3D**). The effect of BEO was significantly antagonized two days after co-administration with naloxone hydrochloride (**Figure 3D**). Immobility time (the threshold value was set at 10 or less per 15 min) in the light phase was not affected by continuous administration of BEO or naloxone hydrochloride (**Figure 3E**). On the other hand, when the threshold value was set to exceed 51 every 15 min, the increase in the two-dimensional planar counts of PSNL-jojoba wax mice during the light phase significantly decreased from day 2 post-surgery of continuous administration of BEO, and continued until the end day of the measurement (**Figure 3F**). The effect of BEO was significantly antagonized four days after co-administration with naloxone hydrochloride (**Figure 3F**).

### Running Wheel Activity in PSNL Mice Decreased in the Dark Phase

**Figure 4:**
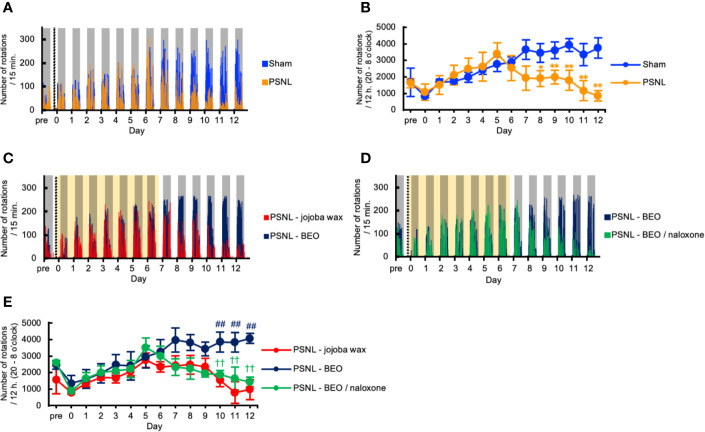
Assessment of running wheel activity in PSNL mice with continuous subcutaneous administration of BEO and naloxone hydrochloride using an osmotic pump. **(A, C, D)** Representative data of running wheel activity in PSNL mice **(A)**, PSNL mice with continuous subcutaneous administration of jojoba-wax and BEO **(C)**, and BEO and BEO/naloxone **(D)** every 15 min using an osmotic pump. **(B, E)** Assessment of running wheel activity in PSNL mice **(B)**, or PSNL mice with continuous subcutaneous administration of jojoba-wax, BEO and BEO/naloxone using an osmotic pump **(E)** during the dark period (20 o’clock–8 o’clock). **(B, E)** Values are the means ± S.E.M. (Sham: n = 7, PSNL: n = 9, PSNL-jojoba wax: n = 6, PSNL-BEO: n = 9, PSNL-BEO/Naloxone: n = 6). **(B)** *P < 0.05; **P < 0.01, compared with Sham and PSNL at corresponding time points (Student’s t-test). **(E)** ^##^P < 0.01, compared with PSNL-jojoba wax and PSNL-BEO at corresponding time points. ^††^P < 0.01, compared with PSNL-BEO and PSNL-BEO/Naloxone at corresponding time points (one-way analysis of variance with Tukey’s test).

We investigated the behavioral phenotype of PSNL mice on the running wheel activity. Representative data showed that the number of rotations in mice with PSNL was decreased in the dark phase from the 8th day after surgery (**Figure 4A**). On the other hand, in the light phase, both sham and PSNL groups rarely rotated the running wheel (**Figure 4A**). The total counts of rotations during the dark phase of PSNL mice decreased significantly from day 8 post-surgery reaching its largest differences at day 12 post-surgery, the final day of measurement (Sham: 3,743.00 ± 610.77, PSNL: 848.78 ± 306.06) (**Figure 4B**).

### Decreased Number of Rotations by PSNL in the Dark Phase Was Abolished by Continuously Administered BEO and Antagonized by Naloxone Hydrochloride

We investigated the effect of the continuous administration of BEO using an osmotic pump on the running wheel activity in PSNL mice. Representative data showed that the decreased number of rotations produced by PSNL in the dark phase was abolished by continuously administered BEO (**Figure 4C**). The effect of BEO on the running wheel activity in mice with PSNL was antagonized by naloxone hydrochloride (**Figure 4D**). Even when the sample was increased, the number of rotations in mice with PSNL-jojoba wax was decreased in the dark phase from the 8th day after surgery. However, this decrease was significantly reduced by the continuous administration of BEO (**Figure 4E**). The effect of BEO was significantly antagonized 2 days after co-administration with naloxone hydrochloride (12 days after surgery; PSNL-jojoba wax: 972.00 ± 543.95, PSNL-BEO: 4,070.33 ± 693.64, PSNL-BEO/Naloxone: 1,431.33 ± 287.24) (**Figure 4E**). These results indicate that the continuous administration of BEO suppressed the decrease in the running wheel activity that was observed in PSNL mice *via* opioid receptors.

### Single Subcutaneous Injection of BEO Suppressed the Increased Two-Dimensional Planar Motion but Did Not Affect the Running Wheel Activity in PSNL Mice

**Figure 5:**
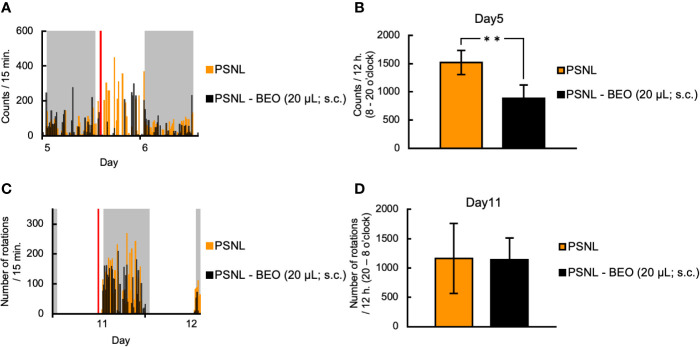
Assessment of behavioral activities in PSNL mice with a single subcutaneous injection of BEO. **(A, B)** Single subcutaneous injection of BEO in PSNL mice was performed at 10 o’clock on the 5th postoperative day. **(A)** Representative data of two-dimensional planar motion every 15 min from 5 to 6 days in PSNL mice with a single subcutaneous administration of BEO. **(B)** Assessment of two-dimensional planar motion during the light period (8 o’clock–20 o’clock) in PSNL mice with a single subcutaneous administration of BEO on the 5th postoperative day. **(C, D)** Single subcutaneous injection of BEO in PSNL mice was performed at 18 o’clock on the 11th postoperative day. **(C)** Representative data of running wheel activity every 15 min from 11 to 12 days in PSNL mice with a single subcutaneous administration of BEO. **(D)** Assessment of running wheel activity during the dark period (20 o’clock–8 o’clock) in PSNL mice with a single subcutaneous administration of BEO on the 11th postoperative day. **(B, D)** Values are the means ± S.E.M. (PSNL: n = 9, PSNL-BEO [20 μl; s.c.]: n = 13). **(B)** **P < 0.01 (Student’s t-test).

Next, we examined whether the behavioral and pharmacological changes induced by the continuous administration of BEO in PSNL mice could be reproduced by a single injection. To examine the effect of a single subcutaneous administration of BEO on a two-dimensional planar movement, the administration was performed at 10 am on the 5th day after surgery. Representative data showed that the increase in the two-dimensional planar motor counts in PSNL mice during the light phase was temporarily suppressed by a single subcutaneous injection of BEO (**Figure 5A**). In the latter half of the light period, the medicinal properties of BEO disappeared and the two-dimensional planar behavior increased again (**Figure 5A**). The total number of the two-dimensional planar counts during the light phase on the 5th day after surgery in PSNL mice was significantly reduced with a single subcutaneous injection of BEO (PSNL: 1,521.44 ± 214.61, PSNL-BEO: 888.77 ± 234.21) (**Figure 5B**).

On the other hand, to examine the effect of a single subcutaneous injection of BEO on the running wheel activity, the injection was performed at 18 o’clock on the 11th day after surgery. Representative data showed that the running wheel activity in the dark phase of PSNL mice was not affected by a single subcutaneous injection of BEO (**Figure 5C**). The total number of rotations during the dark phase of PSNL mice was also unaffected by a single subcutaneous injection of BEO (PSNL: 1,160.50 ± 597.17, PSNL-BEO: 1,145.11 ± 367.81) (**Figure 5D**).

### Inhalation of BEO Did Not Affect the Two-Dimensional Planar Motion and Running Wheel Activity in PSNL Mice

**Figure 6:**
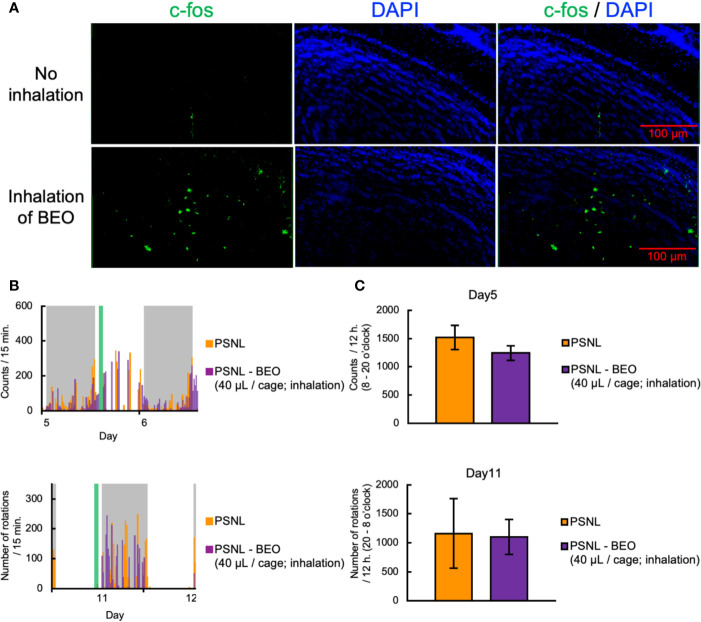
Assessment of behavioral activities in PSNL mice with inhalation of BEO. **(A–C)** Inhalation of BEO by PSNL mice was performed from 10 o’clock to 11 o’clock on the 5th postoperative day. **(A)** Fluorescent immunostaining images of c-fos protein in the olfactory bulb in PSNL mice with inhalation of BEO or no inhalation. **(B)** Representative data of two-dimensional planar motion every 15 min from 5 to 6 days in PSNL mice with inhalation of BEO. **(C)** Assessment of two-dimensional planar motion at the light period (8 o’clock–20 o’clock) in PSNL mice with inhalation of BEO on the 5th postoperative day. **(D, E)** Inhalation of BEO in PSNL mice was performed from 18 o’clock to 19 o’clock on the 11th postoperative day. **(D)** Representative data of running wheel activity every 15 min from 11 to 12 days in PSNL mice with inhalation of BEO. **(E)** Assessment of running wheel activity at the dark period (20 o’clock–8 o’clock) in PSNL mice with inhalation of BEO on the 11th postoperative day. **(C, E)** Values are the means ± S.E.M. (PSNL: n = 9, PSNL-BEO [40 μl/cage; inhalation]: n = 8).

To clarify the behavioral and pharmacological effects of BEO inhalation, we investigated using a double activity monitoring system^®^. To examine the effect of a single inhalation of BEO on two-dimensional planar movement, the mice underwent inhalation for 1 h from 10 am on the 5th day after surgery. To confirm activation of the olfactory signal by inhalation of BEO, olfactory bulbs were sampled from PSNL mice immediately after inhalation of BEO, and fluorescent immunostaining for c-fos, a nerve activity marker, was performed. Representative images of fluorescent immunostaining confirmed that c-fos expression had increased and the olfactory signal was enhanced in this group compared to the group without inhalation (**Figure 6A**). When comparing the two-dimensional planar movement under these conditions, representative data showed that the two-dimensional planar movement in the light period of PSNL mice was not affected by the inhalation of BEO (**Figure 6B**). The total number of two-dimensional planar counts during the light phase on the 5th day after surgery in PSNL mice was not affected by the single inhalation of BEO (PSNL: 1,521.44 ± 214.61, PSNL-BEO inhalation: 1,245.63 ± 129.74) (**Figure 6C**).

On the other hand, to examine the effect of a single inhalation of BEO on the running wheel activity, inhalation was performed at 18 o’clock on the 11th day after surgery. Representative data showed that the running wheel activity in the dark phase of PSNL mice was not affected by a single inhalation of BEO (**Figure 6D**). The total number of rotations during the dark phase of PSNL mice was also unaffected by a single inhalation of BEO (PSNL: 1,160.50 ± 597.17, PSNL-BEO inhalation: 1,103.50 ± 301.09) (**Figure 6E**).

### Inhalation of BEO Did Not Affect Mechanical Allodynia in PSNL Mice

**Figure 7:**
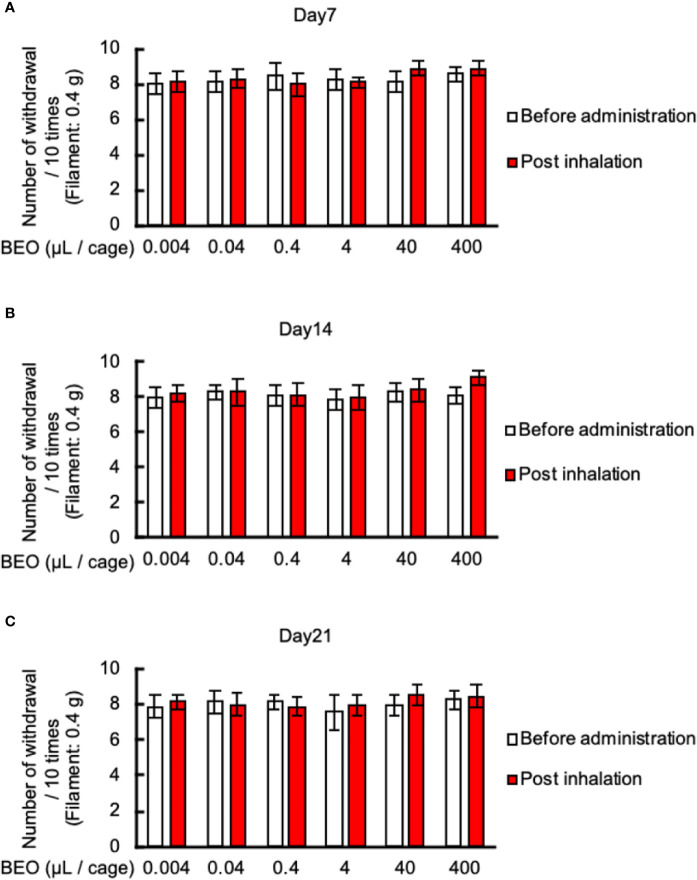
Effects of BEO inhalation on mechanical allodynia in PSNL mice. **(A–C)** Inhalation of BEO by PSNL mice was performed from 10 o’clock to 11 o’clock. After that, mice were removed from the glass cages, and the von Frey test was performed. Effect of BEO inhalation on mechanical allodynia in PSNL mice on the 7th **(A)**, 14th **(B)**, and 21st **(C)** day after PSNL mouse surgery (non-ligation side of Day 7: 1.50 ± 0.23, Day 14: 1.35 ± 0.27, Day 21: 1.61 ± 0.20). Values are the means ± S.E.M. for all experiences (BEO 0.04 μl/cage: n = 8, other groups: n = 7).

Finally, we examined the effect of inhalation of BEO on mechanical allodynia in PSNL mice. The von Frey test showed that inhalation of BEO at 10 o’clock on days 7, 14, and 21 at a dose of 0.004, 0.04, 0.4, 4, 40, 400 μl did not affect mechanical allodynia (**Figures 7A–C**).

## Discussions

This study aimed to elucidate whether the analgesic action of BEO in PSNL mice is mediated by olfactory stimulation by its volatile components. We conducted behavioral pharmacology studies using the double activity monitoring system^®^, which can simultaneously detect the two-dimensional planar motion and running wheel activity at the same time. Besides, BEO was continuously administered subcutaneously with an osmotic pump to eliminate the effects of scent as much as possible.

Few papers regarding behavioral experiments in mice have measured two-dimensional planar motion and running wheel activity at the same time, except for those using the double activity monitoring system^®^. However, many papers have measured each of these independently.

Two-dimensional planar motion is commonly used in “open field tests” and “home cage activity tests” to assess locomotor activity. An open field test is a test that measures spontaneous activity in a new environment (Streng, 1971). On the other hand, a home cage activity test is a test that measures spontaneous activity in the home cage over a medium to long term period (Miyakawa et al., 2003). The ability to capture mouse activity patterns over time can be used to analyze circadian rhythms. Regarding the circadian rhythms of PSNL mice, we reported that the paw withdrawal threshold in PSNL mice fluctuated over about 24 h, with the peak of allodynia persisting from the late light phase to the early dark phase (Kusunose et al., 2010). During the evaluation of the two-dimensional planar motion in this study, using the double activity monitoring system^®^, a peak of abnormal behavior count was observed from 19 o’clock to 20 o’clock, just before the dark period in PSNL mice (**Figure 2B**), which indicates that allodynia symptoms were reflected. On the other hand, the theoretical sustained elimination period of BEO initiated by an osmotic pump at the same time of PSNL surgery is 7 days, and the increased counts of PSNL of the light phase was abolished over the 14-day measurement period (**Figures 2A, D, F****)**. On comparing mechanical allodynia with the von Frey test, the effect of BEO disappeared at 7 days (data not shown in figures). The PSNL mice showed transient mechanical allodynia with the von Frey test, returning to baseline in the sham-operated group at 42 days post-operative, while two-dimensional planar activity did not return to baseline (data not shown in figures). These results suggest that the changes in the two-dimensional planar activity may reflect factors other than allodynia in PSNL mice. Concerning this phenotype, we considered whether sleep is involved. It has been reported that PSNL mice showed decreased non-rapid eye movement sleep (NREM) and sleep duration in the light phase compared to sham-operated mice (Takemura et al., 2011; Wang et al., 2015). Also, certain essential oils, such as bergamot, have been shown to have a hypnotic effect in humans (Lillehei and Halcon, 2014). Therefore, it was considered that the evaluation of the two-dimensional planar motion using the double activity monitoring system^®^ can obtain a phenotype that reflects on sleep and allodynia symptoms.

Regarding the results of two-dimensional planar motion in PSNL mice, the number of counts in the light phase increased (**Figure 2D**) but not in the dark phase (**Figure 2C**). This decrease during the dark period may be due to the rebound effect of increased activity during the light period (sleep period).

The effect of BEO on the two-dimensional planar counts in mice with PSNL was antagonized by naloxone hydrochloride (**Figure 3B**). We performed a continuous administration experiment of the selective μ-opioid receptor antagonist β-FNA and the selective κ-opioid receptor antagonist norbinaltorphimine, instead of the nonspecific opioid receptor antagonist, naloxone hydrochloride. However, at present, it could not be measured well due to the decrease in the number of two-dimensional planar activities during the day and night (sedative effect) (data not shown in figure). In any case, we had previously reported that PSNL mouse-induced allodynia is suppressed by an intra-plantar injection of BEO (Komatsu et al., 2018). This inhibitory effect of BEO on PSNL-induced allodynia is also antagonized by β-FNA but not by naltrindole and norbinaltorphimine (Komatsu et al., 2018). These results suggest that the increased counts produced by PSNL, that were abolished by the continuous administration of BEO in the light phase, may be triggered by activation of peripheral μ-opioid receptors.

The voluntary wheel-running activity is commonly used to assess circadian rhythm or motor function and has been proposed as an observer-independent measure for ongoing pain in inflammatory models (Cobos et al., 2012; Kandasamy et al., 2016). In the neuropathic pain model, it has been reported that the running wheel mileage decreases in spared nerve injury (SNI) model mice (Pitzer et al., 2016), which is almost in agreement with our results in PSNL mice (**Figure 4B**).

We consider that the decrease in the wheel-running activity in PSNL mice was not due to changes in circadian rhythm phases, but due to reduced motivation from chronic pain. Concerning changes in circadian rhythm, the typical wheel-running activity occurred only in the dark phase for both PSNL and sham-operated mice (**Figure 4A**). In the previous report, the expression phase of various clock genes on day 7 after surgery in PSNL mice was unchanged (Koyanagi et al., 2016). This finding supports that reduced running wheel activity in PSNL mice does not reflect changes in the rhythmic phase.

It is widely known that anxiety and depression are secondary to chronic allodynia. A previous report demonstrated that in the 4th week after surgery, PSNL caused significant depression-like behavior in mice, evaluated using the forced swimming test (FST) and the tail suspension test (TST), which was accompanied by increased pain sensitivity (Gai et al., 2014). The delayed appearance of decreased motivation associated with pain may contribute to the explanation that a decrease in running wheel activity in PSNL mice has a time lag from the 8th day after surgery. Our study showed that a single dorsal subcutaneous injection of BEO did not affect the running wheel activity in PSNL mice (**Figures 5C, D****)** and that continuous administration of BEO by osmotic pump corrected the decrease in PSNL mice (**Figures 4C–E**). These results suggest that BEO’s effects on the running wheel activity are mediated by something that is not an opioid receptor but is important in analgesia and two-dimensional planar behavior. It was reported that altered serotonergic (5-HT) neurotransmission is implicated in the antidepressant and anxiolytic properties of physical activity, assuming that the running wheel activity in the double activity monitoring system reflects the pathology of anxiety and depression (Greenwood et al., 2005). We had recently reported that BEO exerts anxiolytic effects by correcting the neuronal activity of the serotonin nervous system *via* the serotonin 5-HT_1A_ receptor (Rombola et al., 2020). In light of the previous reports, continuous administration of BEO did not reduce motivation due to the early analgesic action mediated by opioid receptors, and the anxiolytic action mediated by serotonin 5-HT_1A_ receptors. These effects are considered to be why the reduction in running wheel activity by PSNL mice did not occur after the 8^th^ postoperative day when the release of BEO by the osmotic pump was theoretically completed. On the other hand, a single subcutaneous injection of BEO is expected to increase running wheel activity due to the anxiolytic effect mediated by the serotonin 5-HT_1A_ receptor. However, even in the previous paper, a single injection of BEO did not obtain sufficient anxiolytic effect. Therefore, these findings are consistent with those of the present study in which a single-dose was not efficacious. Based on the above, the running wheel activity in the double activity monitoring system may be decreased in PSNL mice due to anxiety and depression secondary to chronic allodynia pathology. It is considered that recovery by BEO is due to the regulation of the 5-HT_1A_ receptor function.

We have reported that among the constituents of BEO, linalool is important in analgesia (Kuwahata et al., 2009; Sakurada et al., 2009; Sakurada et al., 2011; Kuwahata et al., 2013; Katsuyama et al., 2015). Linalool is one of the major volatile aromatic components contained in BEO. The pharmacokinetics of linalool have been reported to be excreted from urine (approximately 60%), exhaled breath (approximately 23%), and feces (approximately 15%) within 72 h after oral administration of radiolabeled linalool to rats (Parke et al., 1974). The primary metabolite of linalool is in the form of a glucuronide conjugate and is known to be excreted in the feces (Antoine et al., 1993). It is assumed that the metabolites excreted in the feces also have a scent. Therefore, at the beginning of the experiment, it was thought that the scent could be removed by an osmotic pump, but as it is excreted in feces and urine, it is challenging to remove the scent of BEO altogether. However, there are no reports that the metabolites of linalool have an analgesic effect, and it is unlikely that the scents of linalool and linalool metabolites found in manure act in all other complex scents.

In the inhalation experiment of BEO to PSNL mice, we compared the expression of c-fos, a kind of neural activity marker, by fluorescent immunostaining to confirm that the neural activity of the olfactory bulb was activated. The activation occurred only in a part of the olfactory bulb (**Figure 6A**), which was consistent with previous reports (Loch et al., 2013). Generally, when inhaling a scented substance, there are three main routes to be absorbed into the body (Lv et al., 2013). First, it enters the lungs through the respiratory tract and enters the bloodstream through the capillaries of the alveoli. Second, it is absorbed from the capillaries of the nasal mucosa and enters the bloodstream. Third, through a pathway in which it binds to an olfactory receptor of the olfactory cells of the nasal olfactory epithelium, and acts on the central nervous system as an olfactory neurotransmission signal. Of these three routes, the action of the third is produced at the lowest dose. Therefore, in this study, based on the dose (400 μl/cage) used for the reduction of formalin-induced nociceptive behavior by the inhalation of BEO, we investigated every 1/10 up to the dose of 1/100,000 on the low dose side. As a result, no effect of inhalation was found (**Figures 7A–C**) (Scuteri et al., 2018). We consider that by increasing the inhalation dose and exposure time of BEO and increasing its blood transfer, it is possible to confirm the antiallodynic effect, even in inhalation.

## Conclusion

This study suggests that BEO exhibits an analgesic action, which is mediated by opioid receptors but not by the olfactory system. There have been several reports that aromatherapy massage relieves pain in humans. However, our results indicate that at least in mice, olfactory receptors are not involved in the analgesia of severe pain, such as neuropathic pain. Our data also indicates that the running wheel activity in the double activity monitoring system^®^ is useful as a model of anxiety and depression secondary to chronic allodynia.

## Data Availability Statement

The data that support the findings of this study are available from the corresponding author, T.S., upon reasonable request.

## Ethics Statement

The animal study was reviewed and approved by: All experiments were performed following the approval of the Ethics Committee for Animal Experiments at Daiichi University of Pharmacy (Examination number: H30-006, approval number: 29004) and according to the National Institutes of Health Guide for the Care and Use of Laboratory Animals.

## Author Contributions

All authors listed have made a substantial, direct, and intellectual contribution to the work and approved it for publication. KH, SK, TK, KA, DS, GB, and TS designed the research. KH, SK, and TK acquired the data. KA, DS, GB, and TS supplied the experimental materials. KH and KA drafted the manuscript.

## Funding

This study was supported by Grant-in-Aid for Research ActivityStart-up (15H06795; KH) and Grant-in-Aid for Young Scientists (17K18298, 19K16936; KH) from the Japan Society for the Promotion of Science (JSPS), and the Nakatomi Foundation (No. 20181238; KH).

## Conflict of Interest

The authors declare that the research was conducted in the absence of any commercial or financial relationships that could be construed as a potential conflict of interest.
